# Advanced Development of the rF1V and rBV A/B Vaccines: Progress and Challenges

**DOI:** 10.1155/2012/731604

**Published:** 2011-10-17

**Authors:** Mary Kate Hart, George A. Saviolakis, Susan L. Welkos, Robert V. House

**Affiliations:** ^1^DynPort Vaccine Company LLC, Frederick, MD 21702, USA; ^2^Bacteriology Division, USAMRIID, Frederick, MD 21701, USA

## Abstract

The development of vaccines for microorganisms and bacterial toxins with the potential to be used as biowarfare and bioterrorism agents is an important component of the US biodefense program. DVC is developing two vaccines, one against inhalational exposure to botulinum neurotoxins A1 and B1 and a second for *Yersinia pestis*, with the ultimate goal of licensure by the FDA under the Animal Rule. Progress has been made in all technical areas, including manufacturing, nonclinical, and clinical development and testing of the vaccines, and in assay development. The current status of development of these vaccines, and remaining challenges are described in this chapter.

## 1. Introduction

Certain highly pathogenic microorganisms and their products have the potential to be used as weapons against either military or civilian populations. The Centers for Disease Control and Prevention (CDC) classifies these agents into one of three categories (A, B, or C) according to seriousness of consequences following exposure (http://emergency.cdc.gov/agent/agentlist-category.asp; accessed April 7, 2011). The US Department of Defense (DoD) has a long history of developing therapeutics and prophylactics (vaccines) to protect the warfighter against offensive use of these agents. Until relatively recently, these countermeasures could be used under an investigational new drug application (IND) mechanism. Now, the DoD mandates that any such products administered to the US warfighters be licensed by the US Food and Drug Administration (FDA). Currently, DVC is developing two vaccines for DoD's Joint Vaccine Acquisition Program (JVAP); these include a recombinant vaccine to protect against fatal botulism following inhalational exposure to the A1 and B1 serotypes of botulinum neurotoxin (rBV A/B), as well as a recombinant vaccine to protect against pneumonic plague following inhalational exposure to *Yersinia pestis* (*Y. pestis*) (rF1V). The specific performance and regulatory requirements, progress, challenges, and successes in each program are reviewed below.

## 2. Disease Characteristics

### 2.1. Botulism

Botulism is caused by neurotoxins produced by the bacterium *Clostridium botulinum* (*C. botulinum*), and disease presents in different forms including infant, wound, adult colonization, and foodborne botulism [1]. The clinical picture is due to cholinergic inhibition, and characteristic signs include a descending muscle weakness, dry mouth, difficulty swallowing, slurred speech, double or blurred vision, and drooping eyelids. The botulinum neurotoxin (BoNT) eventually causes paralysis of the respiratory muscles, which prevents unassisted respiration and leads to death in a short time. Exposure to aerosolized BoNT leads to another form of disease, known as inhalational botulism, which presents with similar symptoms [2]. Currently, the only points of reference for human lethality following inhalational intoxication are estimates based on DoD modeling and extrapolation from nonhuman primate (NHP) studies (both based on mass of BoNT/70 kg of body weight) and blood levels of BoNT achieved in foodborne cases of botulism. Estimates of human lethality of BoNT serotype A by intramuscular (IM) exposure are within the range of 0.09 to 0.15 *μ*g/70 kg bodyweight and from 0.7 to 0.9 *μ*g/ 70 kg bodyweight by inhalation based on extrapolation from NHP studies [2, 3]. Published estimates of human lethality of BoNT serotype B are not available. The BoNT levels in the sera of patients with botulism are usually less than 10 mouse intraperitoneal lethal dose 50% (MIPLD_50_)/mL [2]. 

Treatment of botulism cases in the USA usually consists of administration of equine antitoxin antisera and supportive care. The equine antitoxins include a licensed bivalent and monovalent antitoxin that contains neutralizing antibodies against BoNT types A/B and E, respectively, and an investigational heptavalent (ABCDEFG) antitoxin. The heptavalent botulinum antitoxin (HBAT, Cangene Corporation) is available through a CDC-sponsored IND protocol. Following expiration of the bivalent and monovalent products in March 2010, HBAT became the only botulinum antitoxin available in the USA for naturally occurring noninfant botulism. Cases of infant botulism are treated with the recently licensed BabyBIG which is derived from the blood of human donors vaccinated with a pentavalent (ABCDE) toxoid vaccine. In NHPs exposed to lethal challenge, treatment is only effective when administered within 18 to 36 hours of exposure.

### 2.2. Plague

Plague is a zoonotic infection with *Y. pestis* that is normally transmitted from rodents to humans when humans are bitten by infected fleas. There are three manifestations of the disease, with flea bites usually causing the bubonic form in which a painful swelling (bubo) of the draining lymph nodes occurs [4, 5]. Left untreated, the infection will cause sepsis and death in approximately half of the cases. Bubo formation is not present in the second form of disease, which leads directly to sepsis, and occurs in about a third of the cases. Bubonic and septicemic infections occasionally progress to secondary pneumonic infections. Infection with *Y. pestis* also rarely occurs through inhalation of the organism, and, after 1 to 6 days, the disease manifests in its pneumonic form, which is nearly always fatal unless the patient is treated with antibiotics within 20 hours of symptom onset [6], and spreads from person to person by respiratory droplets formed during coughing [7–11]. Clinical signs include fever with cough and dyspnea, and there may be production of bloody, watery sputum. Nausea, vomiting, abdominal pain, and diarrhea may also be present.

Other than early diagnosis and treatment of plague with antibiotics, which are essential to survival, there is no licensed plague vaccine available in the United States. The production of a killed, whole-cell plague vaccine (formalin-inactivated *Y. pestis*), previously licensed as *Plague Vaccine United States Pharmacopoeia*, was discontinued in 1999. Moreover, that vaccine was not effective in preventing against primary pneumonic plague.

## 3. DoD Vaccine Performance Requirements

There is a need for vaccines that can protect against respiratory exposure to botulinum neurotoxins and plague. Development of these vaccines by DVC is guided by the DoD performance requirements and demonstration of efficacy in accordance with the Animal Rule (21 CFR 601.91 Subpart H, “Approval of Biological Products When Human Efficacy Studies Are Not Ethical or Feasible”). 

The performance of DoD products is based on user requirements provided in a Capability Development Document (Table 1). For the two vaccines described in this paper, the key performance parameter is FDA licensure. All other requirements are characterized as either threshold (i.e., the absolute minimum acceptable level of performance) or objective (the characteristics that are considered to be ideal). Table 1 lists some of these characteristics for the rBV A/B and rF1V vaccines. These requirements form the basis for assessing the product during its development.

Given that FDA licensure is the ultimate key performance parameter for DoD's medical countermeasures, the overall life cycle development plan is not dissimilar to products under development by the private sector. However, DoD products must also be developed in accordance with acquisition regulations (Federal Acquisition Regulation, Defense Federal Acquisition Regulation Supplement, and others). In addition, these programs are subject to defense funding which is not always as flexible as might be the case with commercial development. 

## 4. Regulatory Strategy: The FDA Animal Rule

Licensure of the rBV A/B and rF1V vaccines will use the FDA Animal Rule. The Animal Rule was established by the FDA to allow development and licensure of products that could not otherwise be licensed using traditional efficacy testing. The Animal Rule may be used for licensure when efficacy studies are unethical due to high pathogenicity, and disease is rare enough in nature that field studies would be either impossible or at least impractical (http://www.fda.gov/OHRMS/DOCKETS/98fr/053102a. htm; accessed April 7, 2011). 

The four major requirements of the FDA's Animal Rule are as follows:

- Requirement no.1: There is a reasonably well-understood pathophysiological mechanism of toxicity of the substance and its prevention or substantial reduction of this toxicity by the product.
- Requirement no.2: The effect is demonstrated in more than one animal species expected to react with a response predictive for humans, unless the effect is demonstrated in a single animal species that represents a sufficiently well-characterized animal model for predicting the response in humans.
- Requirement no.3: The animal study endpoint is clearly related to the desired benefit in humans, generally the enhancement of survival or prevention of major morbidity.
- Requirement no.4: The data or information on the kinetics and pharmacodynamics of the product or other relevant data or information, in animals or humans, allows selection of an effective dose in humans.

**Figure 1:**
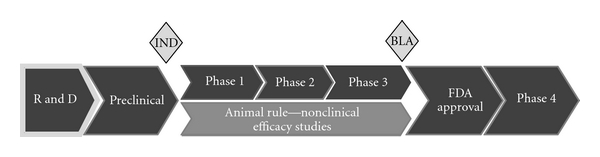
Stages of the advanced development of vaccines. The stages shown in dark grey are common to traditional and FDA's Animal Rule licensure; the additional nonclinical studies are shown in light grey.

As of July 2011, no vaccine has been approved by the FDA under the FDA Animal Rule, so the length of time and costs associated with obtaining approval have yet to be determined. Traditional licensure may take 15 years from discovery to approval; approval of vaccines by the FDA Animal Rule may take longer due to the additional nonclinical requirements (Figure 1) and the need to develop and characterize the animal models and challenge systems. 

## 5. Development of the rBV A/B and rF1V Vaccines

The rBV A/B and rF1V vaccine candidates were initially developed at the US Army Medical Research Institute for Infectious Diseases (USAMRIID). They were transferred to JVAP (and subsequently to DVC) in an early stage of development. The target indication of these vaccines is protection of adults 18 to 55 years of age from disease caused by inhalational exposure to BoNT/A1, BoNT/B1, or *Y. pestis*.

The rBV A/B vaccine candidate [12] comprises the recombinant 50 kDa carboxy-terminal of the heavy chains of Antigen A and Antigen B expressed individually from *Pichia pastoris* using a methanol induction system. Antigen A is derived from the BoNT/A1 expressed by *C. botulinum* strain NCTC 2916 (group I, proteolytic), and Antigen B is derived from BoNT/B1 expressed by *C. botulinum* strain Danish (group I, proteolytic). Antigen A was modified to prevent proteolytic cleavage at the N-terminus during gene expression by removing the codons coding for proteolytically susceptible amino acids. Each 0.5 mL dose of rBV A/B-40 consists of a 1 : 1 mixture of 20 *μ*g Antigen A and 20 *μ*g Antigen B adsorbed to Alhydrogel. 

The rF1V vaccine candidate for plague [13] comprises the F1 capsular protein and the V virulence protein of *Y. pestis* Colorado 92 (CO92) fused into a single protein, which is produced in *Escherichia coli* (*E. coli*) and formulated with Alhydrogel. 

For any product, successful progress towards licensure involves the integration of different functional groups to develop a scalable manufacturing process from the research and development (R&D) proof-of-concept, coordinate the logistics between the release of manufactured material suitable for testing, and stage the appropriate nonclinical, and clinical studies. The progress of the rBV A/B and rF1V vaccines is described below for manufacturing, nonclinical and clinical efforts.

### 5.1. Manufacturing Processes

**Figure 2:**
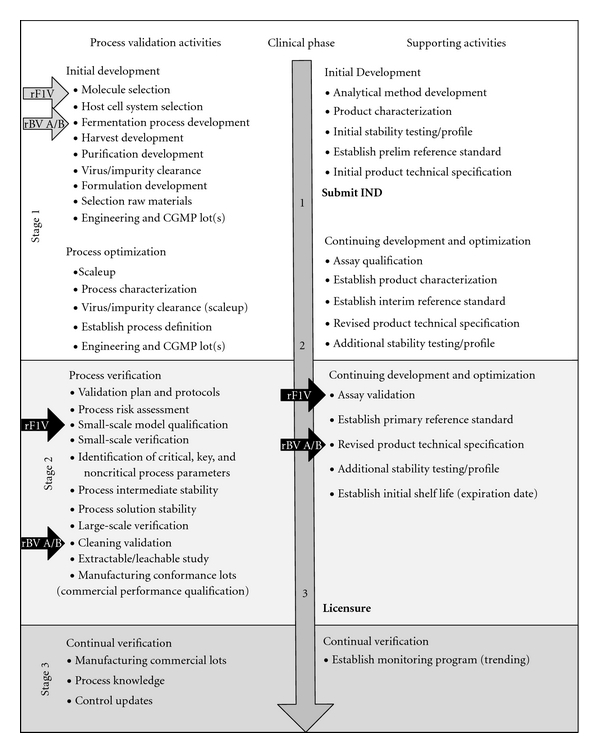
Stages of the manufacturing process, product transition to DVC (grey arrows), and current status (black arrows) of the development efforts for rF1V and rBV A/B vaccines. Some activities may be conducted concurrently.

The rBV A/B and rF1V vaccines were initially developed at USAMRIID and were transferred to advanced development at the stage indicated by the gray arrows in Figure 2. The black arrows indicate the stage of manufacturing development for each product. The products successfully overcame the following challenges to advance to this stage of manufacturing: (1) technology transfer to a contract manufacturing organization and process redesign due to equipment changes, (2) development of manufacturing methods that support the production of clinical trial material and can be validated, (3) scale-up issues associated with either an increase in the number of required troop equivalent doses or nonscalable technological steps, (4) developing needed reagents and analytical methods for product quantitation, purity, and process impurities, and (5) determining conditions that support product stability throughout the manufacturing process. 

The *Pichia pastoris* master and working cell banks for the Antigen A and Antigen B expression strains used to produce the rBV A/B vaccine were generated by expanding accession cell banks produced for each antigen and characterized for purity, identity, and suitability according to the FDA and International Conference on Harmonisation (ICH) guidance. Antigen A and Antigen B are manufactured and adsorbed to Alhydrogel separately and then combined to form the final formulated bulk vaccine. The manufacturing process is at commercial scale (100 L for Antigen A and 600 L for Antigen B). Current Good Manufacturing Practices (CGMP) fill/finish activities were conducted at a 5,000 vial level to support the Phase 1B and Phase 2 clinical trials. Currently the formulated FDP manufacturing process is being scaled to the full commercial scale (approximately 300,000 vials/lot). 

The fused rF1V protein comprising the F1 capsular protein and V virulence protein of *Y. pestis* is produced in *E. coli* and formulated with the adjuvant Alhydrogel. The sequence encoding the rF1V antigen was derived from plasmid pPW731 produced at USAMRIID [13] and was initially expressed from pT5.F1V.1 cell banks but later transitioned to expression from pPW731 cell banks due to intellectual property constraints. Both expression systems use the same regulatory elements for gene expression and the same antibiotic resistance gene for plasmid maintenance. The cell banks were characterized for purity, identity, and suitability according to the FDA and ICH guidance. The manufacturing process was scaled to a final commercial process, which is 1,500 L (working volume for fermentation) and 500 L purification scale. 

Clinical lots for each vaccine were manufactured and released for use in the completed Phase 1 and the ongoing Phase 2 trials, described below. A stability program is ongoing and was designed to establish, maintain, and execute a testing strategy that is compliant with the FDA and ICH guidance.

### 5.2. Nonclinical Studies to Support the Animal Rule Requirements

Animal models are critically important for FDA's Animal Rule licensure, in that they are used to assess vaccine efficacy, and the vaccine-induced animal immune responses are compared to human immune responses to predict clinical benefit. The “Draft Guidance for Industry-Animal Models-Essential Elements to Address Efficacy Under the Animal Rule” released in 2009 was followed to guide the design and execution of nonclinical studies. The nonclinical plans are integrated with the clinical development plans for each vaccine to support the comparison of immune responses across species and to enable the selection of the appropriate human dosage.

The animal studies described below were conducted at accredited facilities under the oversight of an assigned Study Director and attending veterinarian and performed according to the Institute- and the DoD-approved animal protocols. Every effort was made to minimize the suffering and distress of animals exposed to challenge agents or subjected to procedures, using approved anesthetics (1 to 6 mg/kg Telazol for macaques and isoflurane for mice). Biostatisticians were consulted in the study design phase to ensure the study used the proper number of animals needed to achieve interpretable data. Animals were observed multiple times per day for signs of clinical illness during the in-life phase. Macaques were anesthetized and humanely euthanized with an overdose of a euthanasia agent containing pentobarbital when meeting preapproved euthanasia criteria such as decreased body temperature to <93.0°F, >20% loss of body weight from prechallenge weight, respiratory distress/failure, significant reduction in activity (e.g., unable to right itself, complete lack of activity, persistent prostration, or total paralysis), or signs of pneumonia. Mice were euthanized using CO_2_ gas, and guinea pigs were euthanized by a barbiturate overdose injected intraperitoneally or directly into the heart after the animals were anesthetized. 

Proof-of-concept studies conducted by USAMRIID using research material and pilot lots demonstrated the immunogenicity and efficacy of rBV A/B and rF1V vaccines in a variety of animal models including rodents and NHPs. The nonclinical development plans for rBV A/B and rF1V continue the testing in stages: (1) performing initial toxicity studies to support the clinical development, (2) developing and characterizing aerosol challenge models, (3) identifying vaccination regimens that induce immune responses similar to the responses observed in clinical volunteers, (4) demonstrating efficacy in animal models, and (5) conducting final pivotal vaccination/aerosol challenge and reproductive toxicity studies. 

Good Laboratory Practice- (GLP-) compliant nonclinical safety studies were conducted to support clinical testing of the rBV A/B and rF1V vaccines. These evaluated general toxicity following repeat-dose administration in mice and local reactogenicity of administration of a full human dose of vaccine in rabbits. An additional study to evaluate neurobehavioral toxicity was performed for the rBV A/B vaccine. The vaccines produced no apparent systemic toxicity and only mild inflammation at the injection site. The rBV A/B vaccine produced no apparent neurobehavioral toxicity. Together these nonclinical studies supported the initiation of Phase 1 clinical trials. 

An important aspect of developing animal models is the requirement for well-characterized challenge agent. The challenge agents used in the nonclinical efficacy studies are classified as Category A Select Agents by the CDC, and all US facilities that possess or transfer the challenge agent must be registered with the CDC and/or the US Department of Agriculture. The BoNT/A1 and BoNT/B1 were fully characterized to confirm their identity, purity, and strength (biological activity or potency). Protein concentration and biological activity (in terms of MIPLD_50_ units) of both BoNTs were verified using a micro-Bradford protein assay and mouse (toxin) potency assay, respectively. Testing protocols were established to monitor the real time and accelerated stability of the vialed BoNTs. The stability program includes annual testing to confirm the maintenance of strength and purity using the micro-Bradford protein assay, mouse (toxin) potency assay, SDS-PAGE, and size exclusion chromatography. 

The rF1V challenge studies use the CO92 or C12 strains of *Y. pestis*. To ensure the quality and integrity of these strains, challenge material is grown, characterized, and stored in a three-tiered banking system. The banks are characterized by (1) purity on selective media, (2) titer, (3) phenotype, (4) Gram stain, (5) polymerase chain reaction (PCR) for presence of plasmids and chromosomal marker (pathogenicity island), (6) nitrate reduction, (7) antibiotic susceptibility, (8) growth curve, and (9) glycerol fermentation.

The first requirement of the Animal Rule relates to understanding the pathophysiologic mechanism of toxicity and demonstrating that the pathology is similar to that in humans. This is a significant challenge, especially when there is little information available for human disease (e.g., inhalational botulism). Usually studies in two animal species are required for this purpose, unless the disease is well characterized in one animal species and is an accepted model for the human disease. For the rBV A/B and rF1V vaccine development programs, rodent and NHP models were developed to evaluate disease pathophysiology following inhalational exposure of BoNTs and *Y. pestis*. The comparisons of the animal hallmarks of disease to what is known for humans are presented in Tables 2 and 3.

The lethality of BoNT/A1 and BoNT/B1 was determined in CD-1 mice and guinea pigs (unpublished) and recently in rhesus macaques [14]. A stage-wise approach was used to estimate the inhaled median lethal dose (LD_50_) and exposure concentration (LCt_50_). The pathophysiologic responses to aerosol exposure were evaluated for each species to identify relevant endpoints for efficacy studies. The most relevant pathophysiological responses in mice and rhesus macaques were mortality and development of clinical signs of botulism. Clinical observations in all species were consistent with the recognized pattern of botulism disease progression in humans (Table 2). A significant dose response was observed with regard to lethality and the onset and duration of clinical signs in each species. No significant changes in clinical hematology and chemistry and gross and microscopic pathology were observed in mice or rhesus macaques. Changes in physiologic parameters measured by telemetry in rhesus macaques also did not correlate with mortality. 

The pathology induced by *Y. pestis* CO92 was evaluated in animals through clinical chemistries, hematology, telemetry cynomolgus macaques (CMs) only, and detailed histopathology in Swiss Webster mice and CMs. Exposed animals demonstrated multilobar pneumonia, bacterial infiltration of macrophages and lymphoid tissues, fever, sepsis, and death. Data collected from the mouse model development studies estimated the LD_50_ of CO92 to be approximately 2,000 colony-forming units (cfu). 

In the CMs, the inhaled dosage was calculated using the total accumulative tidal volume as measured by plethysmography [15]. The LD_50_ was estimated to be 24 cfu by Probit analysis. Telemetry provided useful information on the clinical course of disease not captured by clinical observations. A rise in temperature routinely coincided with the loss of diurnal rhythm, while increased heart and respiration rate followed by inactivity strongly correlated with a lethal outcome. All CMs with *Y. pestis* positive blood cultures died from pneumonic plague. The pathology in the lungs of all CMs was consistent with the pathology observed in pneumonic plague described in humans. The significant findings are compared across the species in Table 3.

### 5.3. Identification of Vaccination Regimens and Demonstration of Efficacy in Animal Models

The second requirement of the Animal Rule relates to demonstrating that the responses to the countermeasures are similar in animals and humans. The third requirement is to demonstrate the same endpoint in animals that is expected for humans given the vaccine. For the rBV A/B and rF1V vaccines, that endpoint is survival. 

The rBV A/B program vaccinated NHPs with the same material administered to healthy adults in clinical trials and followed the vaccination schedule used for humans. An abbreviated schedule was used for the mice. Neutralizing antibody responses to BoNT/A1 and BoNT/B1 in CD-1 mice and rhesus macaques were evaluated across various vaccine dosages and compared to the neutralizing antibody responses observed in the clinical trial volunteers. Dosages inducing similar antibody levels were identified for both animal models and will be used in pivotal animal studies using the Phase 3 clinical material. 

Initial efficacy studies in rhesus macaques demonstrated protection from aerosol challenge using the identified vaccination regimen. The protective efficacy of the antibody levels induced in humans was assessed using passive transfer studies. A guinea pig passive transfer model was developed and used to demonstrate the protective efficacy of purified immunoglobulin from human rBV A/B vaccinees [17]. 

A similar approach was used for the rF1V program. Vaccine dosage titration studies in CMs and mice are in progress using the material used in the Phase 2b clinical trial to assess the immune responses and efficacy. The first objective is to evaluate survival across five vaccine dosages to select vaccine dosages for use in follow-on studies. The secondary objective is to collect serum from animals in all groups for evaluation in passive transfer studies and to determine the antibody titers in Bridge ELISA (described in more detail below). The follow-on study is designed to confirm the minimum protective dosage of vaccine and estimate the minimum level of antibody in rF1V-vaccinated animals that correlates with surviving aerosol exposure to *Y. pestis *CO92. 

A mouse passive transfer model was developed to assess the ability of immune sera to provide protection from an aerosol challenge. Sera from CMs or human volunteers vaccinated with rF1V were tested in the model and the results described [18]. A definitive correlation between survival in CMs and an antibody level remains to be determined.

The approach taken to meet the Animal Rule requirements is summarized in Table 4 for the rBV A/B and rF1V vaccines and described in more detail below. Briefly, the animal models with similar disease characteristics to those observed in exposed humans are being used to assess the immune responses and efficacy induced by vaccination. The status of progress to date for the specific requirements is indicated in Table 4. The designs and statistical analysis plans for the pivotal GLP nonclinical efficacy studies will need to be prepared and discussed with the FDA. The objective of these studies will be to generate data that supports the Animal Rule requirements to demonstrate efficacy and to extrapolate a dosage likely to produce clinical benefit in humans. These studies will use the Phase 3 clinical trial material, and reproductive toxicity will be assessed concurrently.

### 5.4. Clinical Testing and Human Safety

The rBV A/B and rF1V vaccine candidates were (or are being) tested in the Phase 1 and the Phase 2 clinical trials. All clinical trials were evaluated by a Scientific Merit Review Board consisting of independent experts and approved by an independent Investigational Review Board and the US Army's Human Research Protections Office. The studies were managed by a contracted clinical research organization. Safety was monitored continuously by independent physicians and overseen by a Data Safety Monitoring Board (DSMB). All studies were conducted in accordance with the current Good Clinical Practice as required by applicable US federal regulations (21 CFR Parts 50, 56, and 312) and the ICH guidelines.

Male and female volunteers were recruited and assessed for eligibility after signing an informed consent form. Subjects had to be healthy, as determined by standard screening assessments including medical history, physical examination, and laboratory tests (hematology, chemistries, and urinalysis). For rBV A/B trials, subjects with a history of neurological disorders, immunological disorders or prior therapy with botulinum toxin were excluded. In trials for both vaccines, subjects with a history of use of immunosuppressive drugs, including glucocorticoids, and recent vaccinations were excluded from the study. Study vaccine was administered as a 0.5 mL IM injection in the deltoid muscle. 

Safety monitoring consisted of collection of injection site and systemic reactogenicity data in a volunteer diary via an interactive voice response system after vaccination, and assessment of treatment emergent adverse events (TEAEs) at scheduled and *ad hoc* visits, if needed, throughout the study. Injection site reactions (local reactions) were defined as pain, tenderness, pruritus, redness/erythema, other rash, and swelling or induration. Prespecified systemic reactions included fever, fatigue, myalgia, headache, nausea, vomiting, and diarrhea. Any other system organ manifestation was also to be recorded and evaluated as an adverse event (AE). Grading of AEs was performed by study-specific adaptation of the most up-to-date FDA guidelines for toxicity grading in preventive vaccine clinical trials. Any abnormal laboratory value, abnormal vital sign, or abnormal physical finding that was considered clinically significant by the investigator or met the grading criteria for toxicity of Grade 1 or higher was reported as an AE.

### 5.5. Clinical Trials for rBV A/B

The rBV A/B vaccine was evaluated in adults (18–45 years) in two Phase 1 (rBV A/B-01 and rBV A/B-01B) and one Phase 2 (rBV A/B-02) clinical trials (unpublished). Blood was collected to determine the NAC to BoNT/A1 and BoNT/B1 using the MNA at pre-determined intervals during the study and for calculation of the seroconversion rate. Blood was also collected for passive transfer studies for evaluation of efficacy in animals.

The first trial, rBV A/B-01, was a single-center, open-label, dosage-escalation study designed to evaluate the safety, tolerability, and immunogenicity of a two-dose regimen (Days 0 and 28) of rBV A/B given at three ascending dosages (10 *μ*g, 20 *μ*g, and 40 *μ*g total immunizing protein with adjuvant) and an unadjuvanted antigen-only formulation at the 40 *μ*g total immunizing protein. Forty-four volunteers participated in this study, with 11 in each of the 4 treatment cohorts. The second study, rBV A/B-01B, was a follow-on study to evaluate formulated vaccine administered at two dosages (40 *μ*g and 100 *μ*g total immunizing protein) using four different three-dose schedules (Days 0, 28, 56, Days 0, 28, 112 or Days 0, 28, 182, or Days 0, 56, 182). The addition of a third vaccine dose in the Phase 1B study was expected to increase the level and duration of the immune response. Eighty volunteers participated in this study (10 volunteers per vaccination cohort, 40 volunteers per dosage group). Dose escalation to the higher dosage occurred after a review by the DSMB of all safety data in both studies.

The majority of volunteers experienced at least one TEAE, and most AEs were mild to moderate in intensity and self-limited in both Phase 1 clinical trials. About 30% of the TEAEs were considered related to vaccination, and these generally consisted of injection site reactions, with pain being the most prevalent. Pruritus, erythema, and swelling were reported much less frequently. The most common related systemic reactions included headache, diarrhea, and malaise. Sporadic abnormalities in laboratory test results, most commonly hemoglobin changes, were reported after vaccination in most volunteers but were not considered clinically significant, and there were no notable changes from baseline through the end of each study (6 months after last vaccination) within or across cohorts. There were no serious adverse events (SAEs). The overall incidence of TEAEs and the incidence of administration site reactions were higher in the cohorts that received rBV A/B vaccine compared to the cohorts that received antigens only in the rBV A/B-01 study. No apparent dosage relationship was seen across cohorts that received adjuvanted rBV A/B in either study. 

In the rBV A/B-01 trial, at least 80% of volunteers vaccinated with the two highest dosages (20 *μ*g and 40 *μ*g total immunizing protein) of rBV A/B developed sustained NAC above the lower limit of quantitation for anti-BoNT/A1 and anti-BoNT/B1 antibodies. The antigen-only formulation was not immunogenic. In the rBV A/B-01B trial, administration of three doses of either 40 *μ*g or 100 *μ*g of rBV A/B vaccine elicited detectable levels of neutralizing antibody for both BoNT/A1 and BoNT/B1 in all volunteers. Longer vaccination schedules (third vaccination given at Day 182) elicited a greater NAC than shorter schedules (third vaccination given at Day 56 or Day 112). Based on maximum NAC and antibody kinetics, the vaccination schedule of Days 0, 28, and 182 elicited the highest NAC levels, and there were no significant differences among dosages. 

The rBV A/B-02 Phase 2 trial was a multicenter, blinded, randomized study designed to evaluate the safety, tolerability, and immunogenicity of a three-dose regimen (Days 0, 28, 182 and Days 0, 56, 182) of rBV A/B given at a single dosage of 40 *μ*g total immunizing protein compared to saline placebo. There were 440 volunteers in this study allocated to 2 cohorts of 165 subjects each that received rBV A/B and 2 cohorts of 55 subjects each that received saline. Subjects were followed up to 12 months after the last vaccination. Interim data to 4 weeks after the last vaccination were analyzed; final data analysis is not yet complete.

Nearly all volunteers experienced at least one AE, with approximately the same number among vaccine-treated and placebo-treated volunteers. The majority of AEs were mild or moderate in intensity, and there was no difference in the overall incidence of TEAEs among treatment cohorts. Three subjects were discontinued because of AEs that could be related to vaccination (allergic dermatitis, erythema, and swelling, all at the injection site). There were no SAEs related to study vaccine. More volunteers treated with rBV A/B reported injection site reactions compared to those treated with placebo, the most common being pain, tenderness, swelling, erythema, pruritus, and axillary pain. The most common systemic reactions were headache, myalgia, arthralgia, feeling abnormal, fever, anxiety, malaise, and nausea. Most TEAEs, however, were laboratory values outside the normal range reported as AEs per the protocol. These occurred in about 96% of subjects treated either with rBV A/B or placebo. The most frequently reported laboratory AEs were hemoglobin decrease from baseline or increase from baseline, with no significant difference between treatment cohorts. Most laboratory-related TEAEs were considered mild or moderate in severity and not clinically significant and resolved without treatment. 

The highest neutralizing antibody rates for both anti-BoNT/A1 and anti-BoNT/B1 were observed at Day 210, 28 days after the last vaccine dose and were similar for both vaccination schedules. Final data will evaluate the immune response to one year after last vaccination.

### 5.6. Clinical Trials for rF1V

The rF1V vaccine was evaluated in one Phase 1 (rF1V-01) and one Phase 2 (rF1V-02a) clinical trial. A second Phase 2 (rF1V-02b) clinical trial is ongoing. Male and female volunteers, age 18 to 40 years in the rF1V-01 trial and 18 to 55 years in the rF1V-02a trial, were recruited and assessed for eligibility after signing an informed consent form. These studies were conducted, monitored, and reviewed as described for rBV A/B. The immune response to the vaccine was evaluated by measurement of the concentration of antibodies to rF1, rV, and rF1V by the Bridge ELISA at predetermined intervals during the study and calculation of the seroconversion rate. Blood was also collected for passive transfer studies for evaluation of efficacy in animals.

The first trial, rF1V-01, was a single-center, open-label, dosage-escalation study designed to evaluate the safety, tolerability, and immunogenicity of a two-dose regimen (Days 0 and 28) of rF1V given at four ascending dosages (20 *μ*g, 40 *μ*g, 80 *μ*g, and 160 *μ*g total immunizing protein). Forty-four subjects participated in the study, with 11 per cohort. Based on analysis of the immunogenicity data, an extension study evaluated the effect of a third dose of 160 *μ*g administered about 230 days following the first dose in 8 of 11 subjects who had previously received the same vaccine dosage. All volunteers were followed for 180 days after the last vaccination. 

All volunteers experienced at least one TEAE, and the majority were either mild or moderate in intensity. Injection site reactions were the most frequent related TEAEs and were generally mild or moderate and more frequent at the two highest dosages after two or three vaccinations. The most common injection reactions were pain, swelling, and erythema. Systemic reactions considered related to vaccination were headache, fatigue, nausea, and diarrhea. Most of these reactions were also mild or moderate and were not considered clinically significant. There were no clinically significant or related laboratory changes. After allowance for the different length of time between the vaccinations, there was no apparent increase in the frequency of TEAEs after the second or third vaccination compared to the first vaccination. Serial electrocardiograms were recorded after the first two doses in all cohorts, and no clinically significant abnormalities were observed. 

The rF1V vaccine was immunogenic after two doses of 20 *μ*g, 40 *μ*g, 80 *μ*g, or 160 *μ*g of vaccine. The antibody response was markedly increased in volunteers who received three doses of 160 *μ*g of vaccine, compared to their response after two 160 *μ*g doses. Peak GMCs of all three antibodies tested (anti-rF1, anti-rV and anti-rF1V) occurred 14 days after the third dose. The administration of a third dose also increased the rate of detectable antibody to all three antigens. Based on this study, the two highest dosages (80 *μ*g and 160 *μ*g) were selected for evaluation in the Phase 2a study in a three-dose regimen.

The rF1V-02a Phase 2 trial was a multicenter, blinded study designed to evaluate the safety, tolerability, and immunogenicity of a three-dose regimen (Days 0, 28, and 182 and Days 0, 56, and 182) of rF1V given at two dosages (80 *μ*g and 160 *μ*g total immunizing protein). There were 400 subjects (100 per cohort) in the study. Vaccinated subjects were followed for 12 months after the last vaccination. 

All subjects experienced at least one TEAE, and no statistically significant difference in overall incidence across groups was observed. A total of six volunteers discontinued due to a TEAE, two because of injection site reactions. The majority of TEAEs were mild or moderate in intensity. There were no SAEs related to study vaccine. Most volunteers had TEAEs that occurred within 28 days following a vaccination, and these were primarily injection site reactions. Most of these reactions were mild or moderate in intensity, and the most common were pain, swelling, erythema, and pruritus. The most common related systemic reactions were headache, malaise, nausea, and diarrhea. Most of these reactions also were mild or moderate. The most common laboratory abnormalities reported as AEs were increased blood glucose and protein present in the urine and decreased hemoglobin. These TEAEs were sporadic, not associated with other clinical abnormalities, and resolved without treatment. In general, no clinically meaningful trends were noted in changes to laboratory parameters in any vaccination group, dosage, or schedule. Overall, the 80 *μ*g dosage had a slightly better safety and tolerability profile than the 160 *μ*g dosage, and the Days 0, 56, and 182 schedule had a slightly better safety and tolerability profile than the Days 0, 28, and 182 schedule. In addition, there did not appear to be an increase in the rate of either local or general TEAEs within 28 days after vaccination with subsequent vaccinations.

The immunogenicity data indicate that GMCs for anti-rF1, anti-rV, and anti-rF1V antibodies were much higher after the third vaccination than after the second vaccination, and almost all subjects had evidence of seroconversion 7 to 14 days after the last vaccination. The seroconversion rate was indistinguishable among the selected dosage and schedules following vaccination 3. Both groups that received vaccination on the Days 0, 56, and 182 schedule showed higher anti-rF1, anti-rV, and anti-rF1V GMCs and seroconversion rates from 7 days after the second vaccination to the prevaccination 3 assessment than groups that received vaccination on the Days 0, 28, and 182 schedule. Based on the results of this study, the 80 *μ*g dosage and the Days 0, 56, 182 vaccination schedule were selected for further testing in the Phase 2b clinical trial. In addition, a shorter vaccination schedule is being evaluated in the Phase 2b clinical trial to assess whether or not equivalent immunogenicity is achieved earlier by administering the third vaccination sooner.

In completed human trials, the rF1V vaccine was safe and well tolerated in the dosages and schedules used and elicited an immunological response to the vaccine recombinant antigen (rF1V) and to each of its components (rF1 and rV).

## 6. Plan for Bridging Animal Responses to Predict Human Efficacy

One of the most difficult challenges of licensing vaccines under the FDA Animal Rule is to bridge the animal and human immune responses, demonstrating that the qualitative and/or quantitative immune responses generated in the animal studies are relevant to those observed in humans and can be used to predict clinical benefit and establish an effective dose. This requires a validated assay(s) that serves as a correlate of protection to bridge animal and clinical data. The challenges associated with this become significant if the mechanism(s) of immunity is not well understood, as is the case for plague. 

### 6.1. rBV A/B Program

The protective capacity of antibodies specific for the BoNT was demonstrated in animals [19–23]. Much of the groundwork for elucidating neutralizing antibodies as a correlate of protective immunity was performed by Iakovlev in the 1950s, who demonstrated that passive immunity provided to mice and guinea pigs was sufficient to protect these animals from inhalational challenge of serotype-specific BoNT [24]. The BoNT A/B NACs in sera are assessed in a mouse (toxin) neutralizing antibody assay (MNA) based on the Cardella method [25]. The NACs are generally accepted as a measure of protection from exposure to BoNT.

The results of human studies to date indicate that the rBV A/B vaccine is safe, well tolerated, and immunogenic. The anti-BoNT NACs observed to be protective in animals will be compared to the NACs observed in vaccinated humans to predict clinical benefit in humans (bridging). As noted above, animal vaccine dosages were selected based on their ability to induce NACs that are similar to those obtained in the clinic, and those vaccine dosages and regimens will be used to assess efficacy in pivotal studies. Passive transfer studies designed to be representative of the observed human responses will be an important means of demonstrating the ability of the rBV A/B vaccine to provide protection to humans and for bridging the clinical and nonclinical information. 

### 6.2. rF1V Program

In contrast, there is no accepted assay that correlates with protection from *Y. pestis*. Animals were protected from pneumonic plague following vaccination with the *Y. pestis* F1 and V antigens [26, 27]. F1 is a 17 kDa protein that forms a capsule and may interfere with complement-mediated opsonisation [26, 28]. The 37 kDa V virulence factor is a component of the Type 3 secretion system known as the “Yop virulon.” The Yop virulon induces apoptosis in host phagocytes through the injection of effector proteins from the bacterial cell. The V antigen is secreted in response to environmental stimuli and is critical for virulence [27, 29] independently and as a consequence of its role in facilitating Yop effector translocation.

The F1 and V antigens induce humoral and cell-mediated immune responses in mice, NHPs, and humans [7, 13, 30–38]. The ability of the humoral responses to contribute to protection from bubonic and pneumonic plague was demonstrated with the passive transfer of F1-specific monoclonal antibodies to mice. Likewise, V-specific antibodies protected mice from aerosol challenge with F1+ and F1- strains of *Y. pestis *[39, 40]. More recently, polyclonal and monoclonal antibodies to rF1, rV, or rF1V protected naïve mice from subcutaneous, intranasal, and aerosol exposure to *Y. pestis,* [41, 42] providing additional evidence of a protective role for antibodies. 

Less is known about the role of cell-mediated immune responses in providing protection from plague. Cytokines secreted by T cells, including IFN-*γ* and TNF-*α*, which are believed to activate phagocytes, restrict intracellular *Y. pestis* replication, and facilitate the killing of intracellular bacilli are being studied [37]. In addition, these cytokines appear to contribute to protection by the humoral response, as the neutralization of these cytokines in mice receiving sub-optimal doses of F1- or V-specific antibody significantly reduced survival in these mice [38]. Blocking the cytokine activity with cytokine-specific neutralizing antibodies also interfered with protection in actively or passively vaccinated mice challenged by the respiratory route with *Y. pestis* CO92 [43]. A role for IL-17 in protection from *Y. pestis* is also being investigated [44].

**Figure 3:**
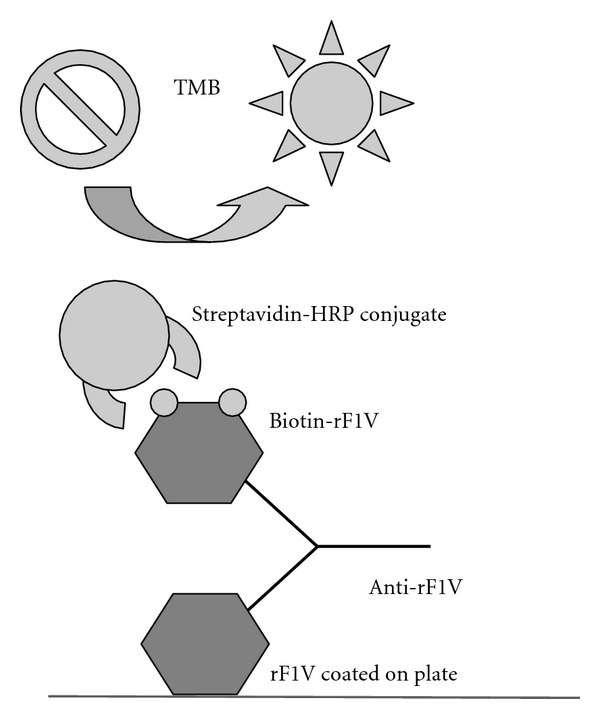
Bridge ELISA schematic.

Bridging the rF1V immune responses to predict clinical benefit is, therefore, more challenging. As humoral responses are known to be involved in the protection from *Y. pestis*, several antibody-based assays are being considered to support bridging. These include a Bridge ELISA (Figure 3), passive transfer of antibodies, and a macrophage cytotoxicity assay.

### 6.3. Bridge ELISA

The Bridge ELISAs use rF1, rV, and rF1V antigens to evaluate the humoral immune response to rF1V vaccination, using a single standard curve that allows direct comparison across clinical and nonclinical samples. The assays are based on the capture of reactive antibodies in immune sera using plates coated with his-F1, his-V, or his-F1V antigen. The bound antibodies are detected by adding diluted biotinylated antigen (rF1, rV, or rF1V) followed by horseradish peroxidase-conjugated streptavidin solutions and tetramethylbenzidine substrate. A chicken IgY standard curve is used to quantitate the levels of antibody to rF1, rV, and rF1V in the tested serum. A representation of the Bridge ELISA for rF1V is presented in Figure 3, and the same format is used for the rF1 and rV assays. 

The key advantage of the Bridge ELISA over a standard direct ELISA is that the same reagents are used across various assays in a manner that has the capacity to be species neutral. The advantage of species neutrality is that it avoids the bias introduced by species-specific secondary reagents. A limitation of the Bridge ELISA is that it is not a functional assay that assesses the protective antibodies in the immune sera but measures any antibody capable of binding the antigen regardless of its protective capacity.

Titers measured in the Bridge ELISA are evaluated statistically for correlation with survival data from direct challenge studies in mice and CMs. Data obtained to date indicate a continuing trend towards correlation with survival, but the data from additional studies will be needed to fully assess the utility of this assay as a correlate. Similarly, the immunogenicity data from the Phase 2b trial will be needed to analyze the human responses in light of the animal titers and efficacy.

### 6.4. Passive Transfer Studies

Passive transfer studies assess the combined protective capacity of antibodies to F1 and V. A mouse passive transfer system was used previously to support a decision in 1941 to vaccinate military personnel under serious threat of exposure to bubonic plague with killed plague bacilli. The readout of the mouse passive transfer assessed both percent mortality and time to death as a ratio termed the Mouse Protection Index (MPI). Based on animal data, MPI values were adopted as a reliable indicator for predicting survival in nonclinical bubonic plague vaccine efficacy studies and to determine when booster vaccinations were required in humans [45, 46].

The passive transfer system involves the administration of immune sera from vaccinated animals or humans to *Y. pestis*-naïve mice. Based on completed pharmacokinetic studies, mice are exposed to aerosol challenge when serum titers plateau. Results of completed CM-to-mouse passive transfer assays demonstrate an association between humoral immunity and protection against pneumonic plague. Results of the survival analysis showed associations of antibody levels with survival although no specific level of circulating antibody (anti-rF1, anti-rV, or anti-rF1V) in either mouse or donor serum has yet been defined as providing a specific level of protection. Mean survival time in the groups of mice that received immune sera was greater than median survival time in the control mice. The survival of the recipient mice, the time to death, and the MPI are being evaluated statistically for correlation with survival in direct aerosol challenge studies in mice and CMs. Human sera from the clinical trials are also being tested and evaluated statistically in both the Bridge ELISA and in the passive transfer system to bridge to the animal data.

### 6.5. Macrophage Cytotoxicity Assay

*Y. pestis* and the other pathogenic species of *Yersinia* are cytotoxic for macrophages and resistant to phagocytosis by cultured macrophages, but the cytotoxicity and resistance can be neutralized by anti-V antibodies. Several macrophage-based assays of immunity to infection by *Y. pestis* were reported [47–51]. 

*Y. pestis* induces macrophage cell death through a caspase-3-dependent apoptotic pathway. One test under active investigation examines the ability of immune serum from rF1V-vaccinated individuals to neutralize *Yersinia- *induced macrophage cytotoxicity by measuring reduction in caspase-3 levels. The key assay components include a mouse macrophage-like cell line, J774A.1, *Y. pseudotuberculosis* (*Y. ptb *[*V*]), where the endogenous V gene has been replaced with the V gene from *Y. pestis*, serum samples from immunized individuals, and the EnzChek caspase-3 II kit. The kit uses a microtiter fluorometric assay and a capsase-3-specific substrate, Z-DEVD-R110, which is cleaved by active caspase-3 to release the highly fluorescent R110. This test is being evaluated for its feasibility as a potential correlate assay. 

Sera from rabbits vaccinated with V or rF1V were first evaluated to verify the ability of immune sera to neutralize macrophage cytotoxicity and to quantitatively detect differences in serum cytotoxicity-neutralizing activity. The cytotoxicity assay was performed at USAMRIID as described in detail previously [48], and a serum neutralization value (NT_50_) determined. The NT_50_ values correspond to the reciprocal of the serum dilution resulting in a 50% decrease in caspase-3 levels. A good dose response was observed in twofold titration assays as determined by regression analysis, and the rabbit antisera yielded NT_50_ values ranging from 157 to 1384; there was no correlation between serum anti-rV ELISA titers and NT_50_ values ([48], data not shown). The results of preliminary evaluations in the macrophage assay of sera from rF1V-vaccinated mice, macaques, and human volunteers were highly suggestive of protection in the animal or passive mouse transfer models.

### 6.6. Remaining Challenges for the rBV A/B and rF1V Development Efforts

The pivotal nonclinical studies and the Phase 3 clinical trials that will be conducted following manufacture of the conformance lots have significant risks associated with the ability to satisfy the FDA's requirements for licensure by the Animal Rule. The most significant technical challenges to be overcome prior to these pivotal studies are successful manufacturing at commercial scale, validation of processes and assays, and identifying a suitable means for predicting clinical benefit for rF1V. In addition to the licensure requirements, the data generated during the advanced development programs will need to satisfy the DoD's performance requirements.

## 7. Summary

Protection of the Nation's warfighters and civilians is crucial to the defense of the United States. One of the threats faced by the warfighter and civilians is the offensive use of biological agents intended to either kill or incapacitate. Advanced development efforts for vaccines against botulinum neurotoxin and pneumonic plague are well on the way toward achieving the performance and efficacy objectives required to advance to pivotal testing prior to applying for FDA licensure.

## Figures and Tables

**Table 1:** Required performance parameters for the rBV A/B and rF1V vaccines.

Key Performance Parameter: FDA Licensure				
Performance attribute	Development threshold		Development objective	
	rBV A/B	rF1V	rBV A/B	rF1V

Efficacy	Protect 80% of immunized persons		Protect 90% of immunized persons	

Immune response	An immune response sufficient to meet threshold efficacy requirements for this vaccine within 210 days of the initial vaccine dose		An immune response sufficient to meet threshold efficacy requirements for this vaccine within 30 days of the initial vaccine dose	

Duration of protection	Protection for at least one year from completion of the primary vaccination series		Protection for five years after the administration of a single-dose vaccine	
Number of doses to achieve protection (primary series)	3		1	

Shelf life	1 year		5 years	

**Table 2:** Symptoms following aerosol exposure to BoNT/A1 or BoNT/B1.

Symptoms	Human^1^	NHP (rhesus macaque)	Guinea pig (Hartley)	Mouse (CD-1)	NHP (rhesus macaque)	Guinea pig (Hartley)	Mouse (CD-1)
		Inhalational BoNT/A1			Inhalational BoNT/B1		
Onset of symptoms: dose dependent	Yes	Yes	Yes	Yes	Yes	Yes	Yes
Lethargy	Yes^1^	Yes	Yes	NR	Yes	Yes	NR
Flaccid paralysis	Yes	NR	Yes	Yes	NR	NR	Yes
Ptosis (drooping eyelids)	Yes	Yes	Yes	Yes	Yes	Yes	No
Dysphagia (difficulty swallowing)	Yes^1^	Yes	NR	NR	Yes	NR	NR
Symmetric, descending paralysis	Yes	Yes	NR	Yes	Yes	NR	NR
Labored respirations	NR^2^	Yes	Yes	Yes	Yes	Yes	Yes
Ataxia	Yes^1^	Yes	NR	NR	Yes	NR	NR
Muscle weakness	Yes^1^	Yes	Yes	Yes	Yes	Yes	Yes
Lateral recumbency	NR	Yes	NR	NR	Yes	NR	NR
Nasal discharge	No	Yes	No?	NR	Yes	Yes	No
Constipation	Yes	Yes	NR	NR	NR	NR	NR
Paresis	Yes	Yes	Yes	Yes	Yes	Yes	NR
Coughing	No	Yes	NR	NR	Yes	No	NR
Piloerection	NR	NR	Yes	Yes	NR	Yes	Yes
Lethality	Yes	Yes	Yes	Yes	Yes	Yes	Yes
Time to death: dose dependent	NR	Yes	Yes	Yes	NR	NR	NR

^1^Specific observations in humans following inhalational exposure.

^2^NR, not reported.

**Table 3:** Clinical signs, gross pathology and histopathology associated with plague infection^1^.

Symptom or lesion	Human	Historical CM	DVC SW mouse	DVC CM
Lymphadenopathy	Yes	Yes	Yes	Yes
Fever	Yes	Yes	ND	Yes
Malaise	Yes	Yes	Yes	Yes
Lethargy	Yes	Yes	Yes	Yes
Elevated pulse	Yes	Yes	ND	Yes
Cyanosis	Yes (late)	Yes	ND	ND
Pharyngitis	Yes	Yes	ND	ND
Cough	Yes	ND	ND	Yes
Rales	Yes	Yes	ND	ND
Sepsis	Yes	Yes	Yes	Yes

Gross pathology primary pneumonic plague				
Fibrinous pleuritis	Yes	ND	ND	Yes
Pneumonia	Yes	Yes	Yes	Yes
Mediastinal hemorrhage	Yes	Yes	ND	Yes
Congestion of trachea/bronchi	Yes	Yes	Yes	Yes

Histopathology primary pneumonic plague				
Pulmonary congestion	Yes	Yes	Yes	Yes
Necrohemorrhagic foci	Yes	Yes	Yes	Yes
Fibrinous pleuritis	Yes	Yes	ND	Yes
Disseminated intravascular coagulation	Yes	Yes	ND	ND
Neutrophil infiltration of lung	Yes	Yes	Yes	Yes
Bacteria in lung	Yes	Yes	Yes	Yes
Mediastinitis	Yes	Yes	Yes	Yes
Bacteria in spleen	Yes	Yes	Yes	Yes^2^

^1^Modified from information contained in Adamovicz and Worsham [16].

CM: cynomolgus macaque, ND: not determined, SW: Swiss Webster.

^2^Bacterial burden not quantitated.

**Table 4:** Vaccine program status for meeting the requirements of the FDA Animal Rule.

Animal Rule	rBV A/B	rF1V
Requirement 1: well understood pathophysiology and amelioration	The published literature has shown that generation of neutralizing antibodies against BoNT provides protection against inhalational botulism.	The published literature has shown that the F1 and V antigens from *Y. pestis* can provide protection from pneumonic plague.
	Pathophysiology following aerosol exposure of CD-1 mice and rhesus macaques is comparable to the pathophysiology of disease in humans.	Pathophysiology following aerosol exposure of Swiss Webster mice and CMs is comparable to the pathophysiology of disease in humans.
	Vaccination with rBV A/B elicits a humoral immune response in mice and macaques that provides protection against exposure to aerosolized neurotoxins.	Vaccination with rF1V elicits a humoral immune response in mice and macaques that provides protection against exposure to aerosolized *Y. pestis*.

Requirement 2: effect is demonstrated in more than one species	The mouse and macaque models have immune responses to vaccination with rBV A/B that are similar to the response in humans. Data obtained to date indicate that vaccination induces neutralizing antibody titers believed to be protective in tested species.	The Swiss Webster mouse and cynomolgus macaque models have immune responses to vaccination with rF1V that are similar to the response in humans. Data obtained to date indicate that antibody titers to F1 and V are induced in tested species.

Requirement 3: the animal study endpoint is related to the desired benefit in humans	Nonclinical efficacy study endpoints measure survival against an aerosol challenge, which is the desired benefit in humans.	Nonclinical efficacy study endpoints measure survival against an aerosol challenge, which is the desired benefit in humans.

Requirement 4: data allows selection of an effective dose in humans	The mouse toxin-neutralizing antibody assay (MNA) provides a species-neutral assay for quantitating the level of neutralizing antibodies.	The Bridge ELISAs are in development as species-neutral assays that permit direct comparison across samples from different species.
	The neutralizing antibody concentration (NAC) determined by the MNA is under evaluation as a correlate of protection.	Bridge ELISA, macrophage cytotoxicity assays, and passive transfer studies are under evaluation for correlation with protection.
	Passive transfer assesses the protective capacity of antibodies present *in vivo* at the time of aerosol challenge. This is under development as a model to assess the protective capacity of transferred immunoglobulin from human vaccines.	Passive transfer assesses the protective capacity of antibodies present *in vivo* at the time of aerosol challenge. This is under development for consideration as a model to assess the protective capacity of transferred serum from human vaccines.
